# Mitochondrial Fragmentation in *Aspergillus fumigatus* as Early Marker of Granulocyte Killing Activity

**DOI:** 10.3389/fcimb.2018.00128

**Published:** 2018-05-14

**Authors:** Dominik Ruf, Victor Brantl, Johannes Wagener

**Affiliations:** ^1^Max von Pettenkofer-Institut für Hygiene und Medizinische Mikrobiologie, Medizinische Fakultät, LMU München, Munich, Germany; ^2^Institut für Hygiene und Mikrobiologie, Julius-Maximilians-Universität Würzburg, Würzburg, Germany

**Keywords:** *Aspergillus fumigatus*, killing, assay, PMNs, granulocytes, mitochondria, mitochondrial morphology, fungicidal activity

## Abstract

The host's defense against invasive mold infections relies on diverse antimicrobial activities of innate immune cells. However, studying these mechanisms *in vitro* is complicated by the filamentous nature of such pathogens that typically form long, branched, multinucleated and compartmentalized hyphae. Here we describe a novel method that allows for the visualization and quantification of the antifungal killing activity exerted by human granulocytes against hyphae of the opportunistic pathogen *Aspergillus fumigatus*. The approach relies on the distinct impact of fungal cell death on the morphology of mitochondria that were visualized with green fluorescent protein (GFP). We show that oxidative stress induces complete fragmentation of the tubular mitochondrial network which correlates with cell death of affected hyphae. Live cell microscopy revealed a similar and non-reversible disruption of the mitochondrial morphology followed by fading of fluorescence in *Aspergillus* hyphae that were killed by human granulocytes. Quantitative microscopic analysis of fixed samples was subsequently used to estimate the antifungal activity. By utilizing this assay, we demonstrate that lipopolysaccharides as well as human serum significantly increase the killing efficacy of the granulocytes. Our results demonstrate that evaluation of the mitochondrial morphology can be utilized to assess the fungicidal activity of granulocytes against *A. fumigatus* hyphae.

## Introduction

*Aspergillus fumigatus* is a filamentous fungus and major opportunistic human pathogen. This mold is found in most cases of aspergillosis, a wide variety of diseases caused by *Aspergillus* species (Denning, 1998; Kousha et al., 2011; Kosmidis and Denning, 2015). *A. fumigatus* spreads with airborne asexual spores, the conidia. Due to the ubiquitous environmental distribution of *Aspergilli* humans inhale several hundred conidia per day. In healthy individuals these inhaled conidia and eventually forming *Aspergillus* hyphae are controlled and rapidly inactivated by alveolar macrophages and neutrophil granulocytes (polymorphonuclear leukocytes; PMNs). However, in patients that suffer from severe immunodeficiency, e.g., those treated with steroids or for hematooncologic malignancies, *Aspergillus* hyphae can unimpededly invade the tissue and subsequently spread to other organs. Consequently, neutropenia and phagocyte dysfunctions, such as chronic granulomatous disease (CGD), are major risk factors for invasive aspergillosis (IA) (Kousha et al., 2011; Kosmidis and Denning, 2015). The mortality of IA is extraordinarily high and ranges from 30 to 95% (Brown et al., 2012).

Cells of the innate immunity have a key role in the defense against invasive aspergillosis and many previous and ongoing studies focus on this aspect. One of the most important challenges in related experimental studies is a quantitative analysis of the killing efficacy of immune cells against the pathogen. Various sophisticated methods have been established to quantify the killing activity of immune cells. Killing efficacy against unicellular microbes (e.g., single bacteria, yeasts or spores) is often determined by plating on agar plates and counting the surviving microorganisms as colony-forming units. With filamentous multicellular and multinucleated fungi such as *A. fumigatus* the situation is more complicated. Counting colony-forming units after killing does not commensurably correlate with the viability of the compartmentalized *Aspergillus* hyphae and microcolonies. Alternative experimental approaches therefore primarily rely on measuring the metabolic activity after killing with colorimetric tetrazolium reduction assays (for example XTT or MTT assays) and comparing it to untreated controls (for example: Bianchi et al., 2009; Lee et al., 2015; Loures et al., 2015; Gazendam et al., 2016b). However, these assays are indirect and do not differentiate between killing and growth inhibition (e.g., as exerted by neutrophil extracellular traps (NETs) (Bruns et al., 2010; McCormick et al., 2010; Gazendam et al., 2016c). Moreover, comparison of *Aspergillus* wild type and respective mutants can be hampered by possible mutation-dependent differences in metabolism. Recently, FLuorescent *Aspergillus* REporter (FLARE) conidia were introduced and utilized to quantify the killing activity of monocytes and neutrophils against *A. fumigatus* conidia (Jhingran et al., 2012, 2016; Espinosa et al., 2014; Brunel et al., 2017; reviewed in Heung et al., 2015). The concept of this approach relies on fading of cytosolic fluorescence proteins after killing in the phagolysosome. However, if rapidly replicating and potentially extracellular morphotypes (e.g., *Aspergillus* hyphae) are studied, the degradation kinetics of such fluorophores can be critical (discussed in Heung et al., 2015).

Here, we report a new microscopy-based method that combines fading of fluorescence proteins with cell death-associated alterations in the cellular architecture to quantify the killing activity of human granulocytes against *A. fumigatus* hyphae. The method relies on the distinct effects of cytotoxic conditions on the morphology and dynamics of mitochondria within *Aspergillus* hyphae. Following the concept of FLARE we named our approach Mitochondria and FLuorescence *Aspergillus* REporter (MitoFLARE). By using this assay, we demonstrate that human serum as well as lipopolysaccharides significantly increase the killing efficacy of human granulocytes against the mold. In combination with metabolism-based assays our assay allows for the discrimination of growth inhibitory and cytotoxic antifungal effects.

## Materials and methods

### Strains, culture conditions, and chemicals

The non-homologous end joining-deficient strain AfS35, a derivative of D141, was used as wild type in this study (Krappmann et al., 2006; Wagener et al., 2008). Mitochondrial morphology in *Aspergillus* hyphae was visualized with mitochondria-targeted green fluorescent protein (GFP). To this end, AfS35 was transformed with pCH005 and D141 with pRS54-phleo, essentially as described before (Neubauer et al., 2015). pCH005 and pRS54-phleo both encode an N-terminal mitochondrial targeting signal, the first 59 amino acids of the *Aspergillus niger* citrate synthase fused to the coding sequence of a GFP derivative (sGFP) under control of the constitutive *Aspergillus nidulans gpdA* promoter. The cytosolic sGFP-expressing strain was constructed by transforming AfS35 with pJW103 (Dichtl et al., 2010). Strains were raised on *Aspergillus* minimal medium (AMM) (Hill and Kafer, 2001) to obtain and harvest conidia. Experiments were performed in RPMI-1640 medium (11835-063; Gibco, Thermo Fisher, Waltham, Massachusetts) or AMM, as indicated. All experiments performed in RPMI-1640 were incubated with 5% CO_2_. Resazurin (R7017) and calcofluor white (F3543) were obtained from Sigma Aldrich (St. Louis, MO, USA), hydrogen peroxide (H_2_O_2_) was obtained from Carl Roth (8070.2; Karlsruhe, Germany) and Percoll was obtained from GE Healthcare (10253000; Uppsala, Sweden). Lipopolysaccharides (LPS) were purchased from Invivogen (tlrl-peklps; San Diego, CA, USA).

### Isolation of human granulocytes

Granulocytes were isolated from blood of healthy adult volunteers who gave informed written consent. Collection was conducted according to the Declaration of Helsinki and was approved by the Ethics Committee of the LMU München. Heparinized peripheral blood was collected in tubes. Autologous plasma was obtained from one tube by centrifugation (1,000 g, 10 min) and layered over the blood in the remaining tubes (~0.8–1 ml plasma on 7.5 ml blood per tube). To separate red from white blood cells, blood was then allowed to sediment for ~90 min at room temperature. 3–5 ml of the supernatant containing leukocytes was then transferred to new tubes on top of a Percoll gradient (4 ml 55% (v/v) in PBS and 3 ml 74% (v/v) in PBS, at room temperature). Tubes were subsequently centrifuged for 22 min at 600 g. After centrifugation, granulocytes were located in the phase between the two percoll layers. Granulocytes were transferred to a new tube and washed with PBS (300 g, 10 min). The viability and cell count was determined with the trypan blue exclusion method (>97%) in a hemocytometer.

### Quantification of metabolic activity with resazurin

The metabolic activity of *Aspergillus* hyphae after exposure to H_2_O_2_ or granulocytes was analyzed with a resazurin reduction assay over time (Monteiro et al., 2012). 1.5 × 10^4^ conidia were inoculated in 100 μl RPMI-1640 per well and cultivated at 37°C with 5% CO_2_. After a total incubation of 10 h, 1.5 × 10^6^ granulocytes resuspended in 100 μl pre-warmed RPMI-1640 or 100 μl pre-warmed medium without granulocytes or H_2_O_2_ in different concentrations were added per well. When indicated 10% (v/v) human serum or an equal volume of medium was added 30 min prior to addition of granulocytes. In case of granulocyte killing experiments, medium was discarded after 2 h incubation and replaced with 100 μl ice-cold ddH_2_O to lyse non-fungal cells (e.g., granulocytes) or, as lyzed-granulocytes-control, replaced with 100 μl ice-cold ddH_2_O with 1.5 × 10^6^ granulocytes. After 30 min incubation at room temperature, 100 μl 2 × AMM with a final concentration of 0.002% (w/v) resazurin was added. In case of H_2_O_2_ killing experiments, medium was discarded after 2 h incubation and replaced with RPMI-1640 supplemented with 0.002% (w/v) resazurin. Plates were subsequently incubated and analyzed over time in a BMG Labtech CLARIOstar microplate reader (excitation: 550–15 nm, dichroic: 568.8, emission: 590–20 nm, excitation and detection from top; BMG Labtech, Ortenberg, Germany).

### Microscopy-based evaluation of anti-hyphal killing efficacy

Experiments were performed in μ-Slide 8 Well slides (#80826; Ibidi, Martinsried, Germany). 3 × 10^3^ conidia expressing mitochondria-targeted GFP were inoculated in 300 μl RPMI-1640 per well and cultivated at 37°C with 5% CO_2_. After a total incubation of 10 h, 1.5 × 10^6^ granulocytes resuspended in 100 μl pre-warmed RPMI-1640 or 100 μl pre-warmed medium without granulocytes with or without the indicated amount of H_2_O_2_ were added per well. When indicated, samples were fixed with paraformaldehyde and stained with calcofluor white after the indicated incubation time. To this end, medium was discarded and samples were fixed with 4% paraformaldehyde for 10 min. When indicated, hyphae were subsequently stained with 1 mg ml^−1^ calcofluor white in ddH_2_O for 10 min. Fixed and stained samples were washed with PBS. The mitochondrial morphology of the hyphae was analyzed with fluorescence microscopy as described below.

### Quantitative analysis of mitochondrial morphology

Quantitative analysis of the mitochondrial morphology was performed with masked and randomized samples. Three samples were analyzed per condition and each experiment. The mitochondrial morphology of 60 hyphae per sample was examined and directly evaluated with an inverted fluorescence microscope using a 63x objective with immersion oil. To this end, hyphae were evaluated in consecutive fields of view. In each field of view, hyphae exposing GFP fluorescence were first localized and visualized using a conventional GFP fluorescence filter cube. The sizes of hyphal compartments as well as hyphae and hyphal compartments that did not expose GFP fluorescence were subsequently analyzed based on the calcofluor white fluorescence using a conventional DAPI fluorescence filter cube. Vital hyphae were defined as single hyphae exhibiting tubular or partially tubular mitochondria in compartments that encompass more than 40% of the hyphal volume. Hyphae exhibiting no fluorescence, fading fluorescence or complete fragmentation of the mitochondrial morphology with or without clustering in compartments encompassing equal or more than 60% of the hyphal volume were defined as not vital or significantly affected in viability. The killing efficacy of each batch of isolated granulocytes was evaluated with an unblinded control sample prior to the full examination of a masked and randomized experiment. To allow for the comparison of killing efficacy under different conditions, experiments demonstrating excessive or no significant killing efficacy of the isolated granulocytes (hyphal vitality after killing for 2 h in the absence of LPS and serum: <30% or >90%) were excluded and not considered in the subsequent statistical analyses in this study. On that score, of 11 experiments one (9%) was excluded because of too high killing activity and two (18%) because of too low killing activity. Statistical significance (^***^p ≤ 0.001; ^**^p ≤ 0.01; ^*^p ≤ 0.05) was calculated with a two-tailed unpaired (assuming unequal variances) Student's *t*-test or the one-way ANOVA analysis of variance with Tukey's multiple comparison post-test as indicated in the figure legend. Analysis was done in GraphPad Prism software (V.5).

### Microscopy

Fluorescence microscopy was performed with a Leica SP5 inverted confocal laser scanning microscope (Leica Microsystems, Mannheim, Germany) equipped with a climate chamber (The Cube & The Box, Life Imaging Services, Switzerland) and a gas mixer (The Brick, Life Imaging Services) to obtain images or with a Leica DM IRB inverted microscope (Leica Microsystems) for direct quantitative analysis. Live cell imaging was performed at 37°C and with 5% CO_2_ if RPMI-1640 was used. The mitochondrial morphology in *Aspergillus* hyphae was visualized by expressing mitochondria-targeted GFP. A 63x objective with immersion oil was used for a quantitative evaluation of mitochondrial morphology. When indicated, samples were fixed with 4% paraformaldehyde for 10 min and stained with 1 mg ml^−1^ calcofluor white dissolved in ddH_2_O for 10 min and subsequently washed with PBS as described above. Experiments were performed in μ-Slide 8 Well slides (#80826; Ibidi) for fluorescence microscopy or in 24 or 96-well cell culture plates for bright and dark field microscopy. Image processing was performed with the LAS AF software (Leica Microsystems). Conventional bright and dark field images were taken with an EOS 550D digital camera (Canon, Tokyo, Japan) fitted to an Axiovert 25 inverted microscope (Carl Zeiss MicroImaging, Göttingen, Germany).

## Results

### Oxidative stress induces non-reversible fragmentation of the mitochondrial network

**Figure 1:**
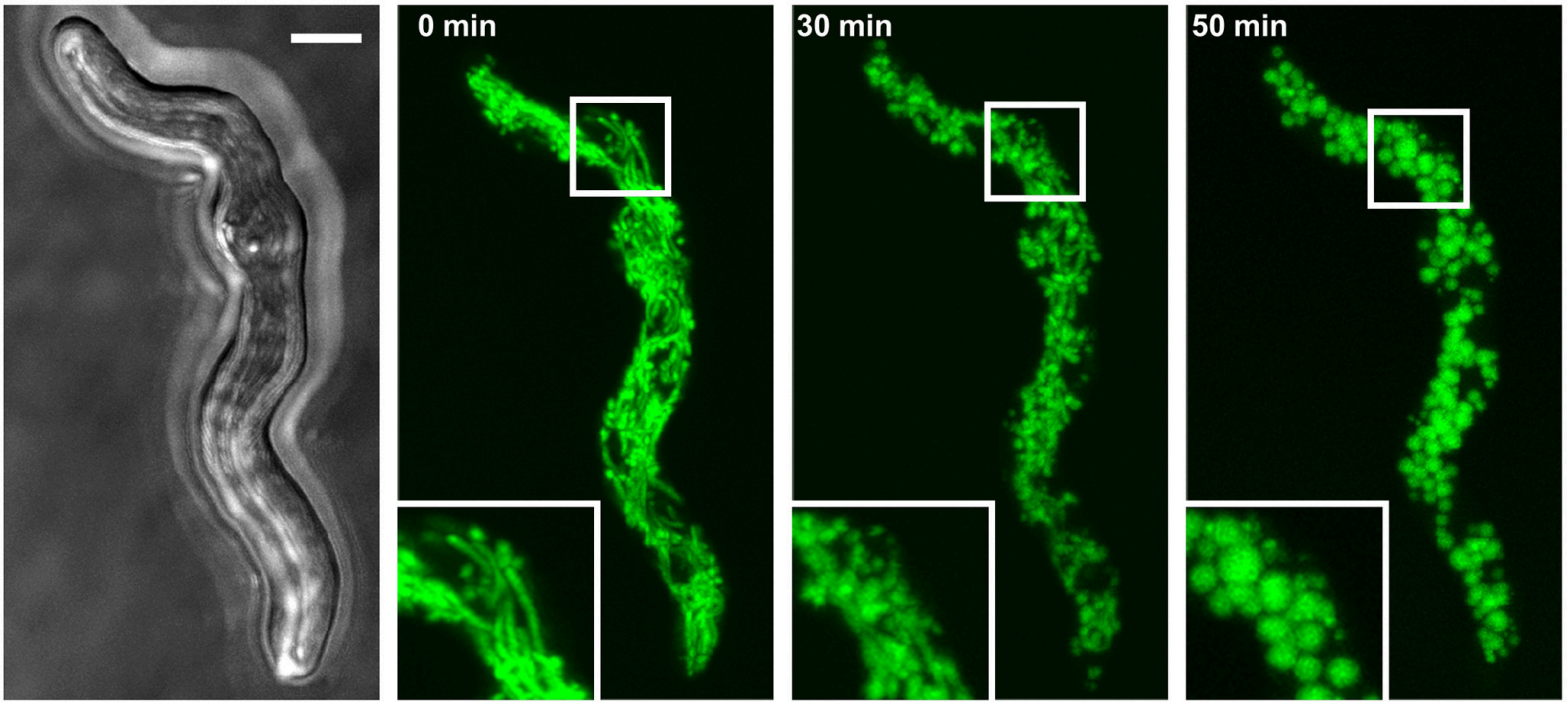
Hydrogen peroxide induces fragmentation of the mitochondrial network. *A. fumigatus* conidia expressing mitochondria-targeted GFP were inoculated in AMM. After 10 h incubation at 37°C, medium was supplemented with 3 mM H_2_O_2_. The mitochondrial morphology of the depicted hyphae was documented over time with confocal laser scanning microscopy. An exemplary bright field image **(left)** and time-lapse GFP fluorescence images of optical stacks covering the entire hyphae in focus after 0, 30, and 50 min exposure (green; **middle** and **right**) are depicted. The bar represents 4 μm.

**Figure 2:**
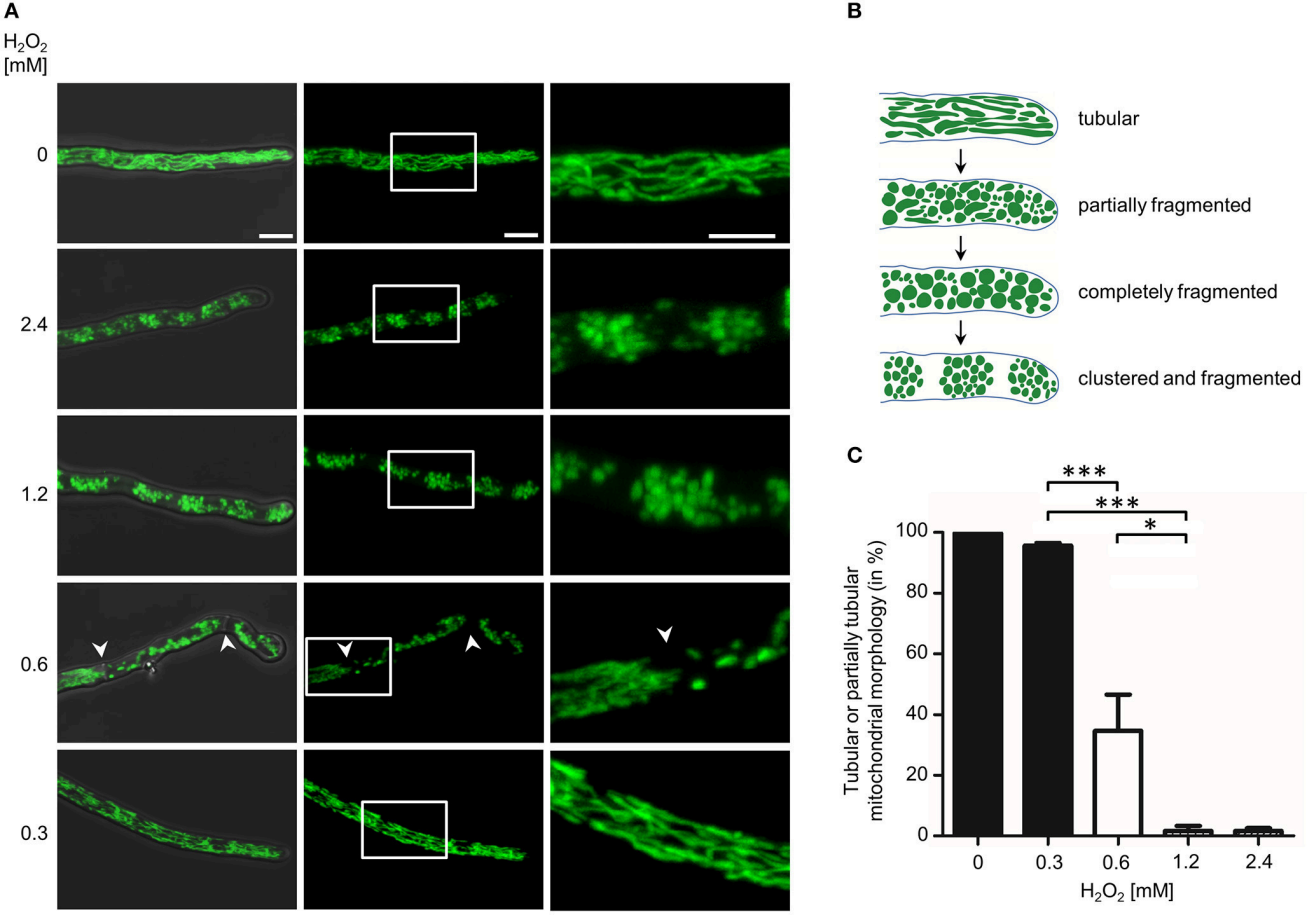
Impact of different hydrogen peroxide concentrations on the mitochondrial morphology in *A. fumigatus* hyphae. **(A,C)** *A. fumigatus* conidia expressing mitochondria-targeted GFP were inoculated in RPMI-1640 and incubated at 37°C. After 10 h, medium was supplemented with the indicated amount of H_2_O_2_. After 2 h incubation, samples were fixed and analyzed with fluorescence microscopy. **(A)** Representative bright field and GFP fluorescence images of optical stacks covering the entire hyphae in focus were taken with a confocal laser scanning microscope. Overlays of bright field and fluorescence images (left panels), fluorescence only (middle panels) and magnifications of the framed sections (right panels) are depicted. Bars represent 5 μm and are applicable to all respective subpanels below. The arrowheads indicate septa that separate distinct hyphal compartments with tubular or fragmented mitochondrial morphology. **(B)** Cartoon of tubular, partially fragmented and partially tubular, completely fragmented and completely fragmented and clustered mitochondrial morphology. **(C)** The ratio of hyphae with tubular or partially tubular mitochondrial morphology in more than 40% of a hyphal volume was determined as described in the material and method section. Statistical significance (^***^p ≤ 0.001; ^*^p ≤ 0.05) was calculated with a two-tailed unpaired (assuming unequal variances) Student's *t*-test. The error bars indicate standard deviations.

Mitochondria form tubular and highly dynamic networks in *A. fumigatus* hyphae (McCormick et al., 2010; Neubauer et al., 2015; Wagener, 2016; Geißel et al., 2017). As part of our studies, we observed that cytotoxic conditions cause characteristic and non-reversible changes in the mitochondrial morphology combined with an arrest in mitochondrial dynamics in *Aspergillus* hyphae. For example, exposure to fungicidal concentration of hydrogen peroxide (H_2_O_2_) rapidly induce fragmentation of the normally tubular mitochondrial network in less than 1 h (Figure 1). Importantly, neither the H_2_O_2_ susceptibility of *A. fumigatus* nor the H_2_O_2_-induced changes in mitochondrial morphology and dynamics depended on non-homologous end joining, a DNA double-strand break repair machinery that is disrupted in many laboratory strains for a more efficient yield of mutants (Supplementary Figure 1). To further investigate this phenomenon, we experimentally delimited the H_2_O_2_ concentration range that reproducibly induces mitochondrial fragmentation (Figure 2). H_2_O_2_ concentrations of 1.2 mM and higher caused complete fragmentation of the mitochondrial network in almost all exposed hyphae (>95%) within 2 h. Besides fragmentation of the mitochondrial network, we additionally observed the accumulation of mitochondrial clusters in many hyphae (Figures 2A,B). In a limited number of hyphae (<10%) no or almost no fluorescence was detectable. This was in good agreement with persisting fluorescence in the vast majority of hyphae expressing cytosolic GFP under similar conditions (Supplementary Figure 2). H_2_O_2_ concentrations of 0.3 mM and below had no obvious impact on the mitochondrial morphologies in the *Aspergillus* hyphae. 0.6 mM H_2_O_2_ induced mitochondrial fragmentation in many but not all hyphae. Interestingly, some hyphae presented segments with fragmented mitochondrial morphology directly adjacent to a segment with tubular mitochondrial morphology. A closer examination revealed that such segments were separated by hyphal septa (see arrows in Figure 2A, 0.6 mM H_2_O_2_). Based on our observations we quantified the impact of these different H_2_O_2_ concentrations on the mitochondrial morphology. Because some hyphae showed segments with tubular and non-tubular mitochondrial morphology (Figure 2A, 0.6 mM H_2_O_2_), only the hyphae exhibiting completely fragmented mitochondrial morphology or fluorescence fading in the majority of the hyphal volume (approx. more than 60%) were counted as significantly affected. As shown in Figure 2C, 1.2 mM H_2_O_2_ and higher caused complete fragmentation or disappearance of fluorescence in almost all hyphae while concentrations equal to or below 0.3 mM H_2_O_2_ hardly affected the mitochondrial networks. Exposure to 0.6 mM H_2_O_2_ caused complete fragmentation or disappearance of fluorescence in 60–70% of the hyphae.

### Mitochondrial fragmentation correlates with cell death of individual hyphae

**Figure 3:**
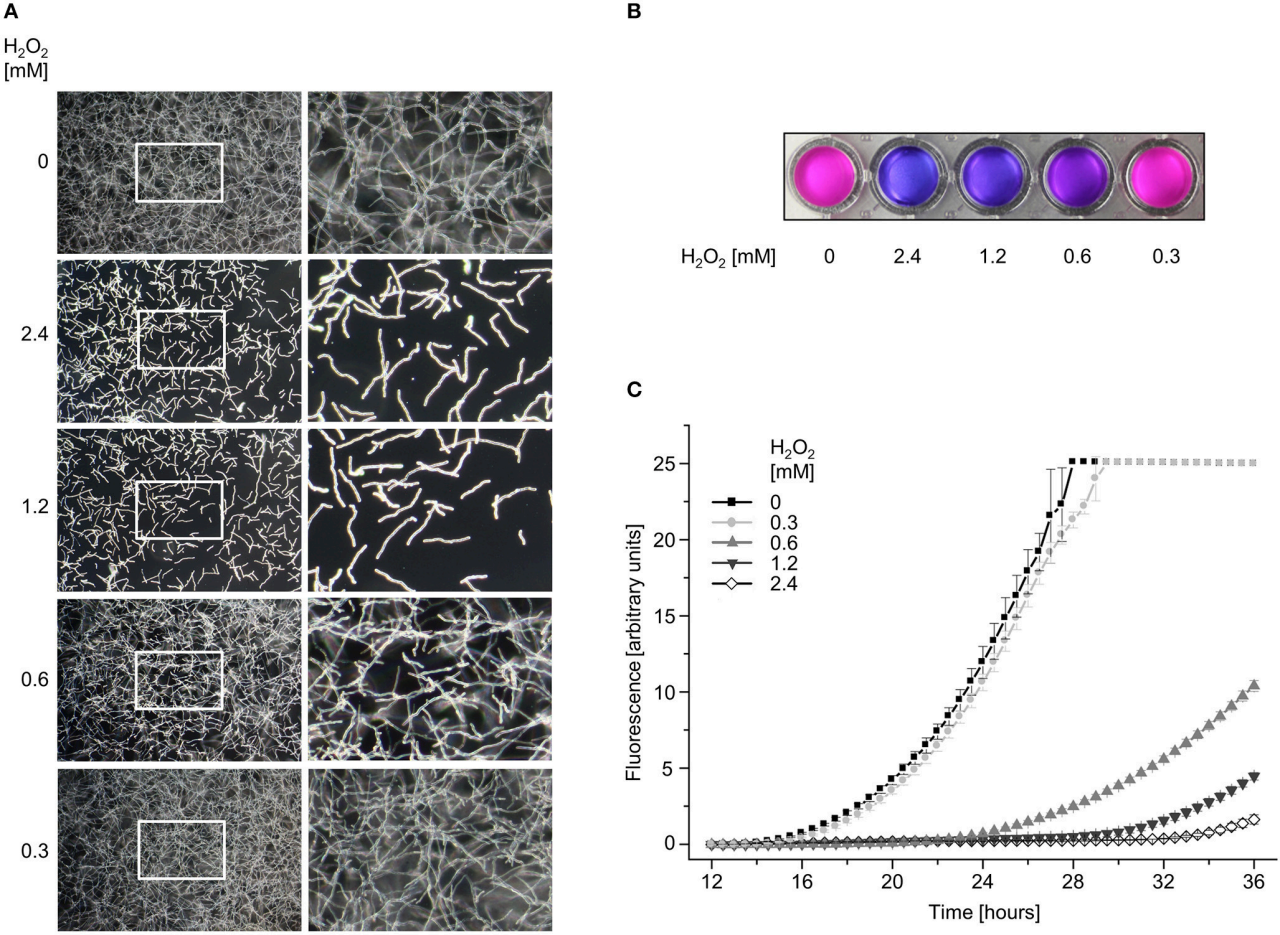
The viability of *A. fumigatus* hyphae correlates with the hydrogen peroxide-induced alterations in the mitochondrial morphology. **(A)** *A. fumigatus* wild type conidia were inoculated in RPMI-1640 in a 96-well plate and incubated at 37°C. After 10 h, medium was supplemented with the indicated amount of H_2_O_2_. After 2 h incubation, media were replaced with RPMI-1640 without H_2_O_2_. After 12 h further incubation, the depicted representative dark-field images were taken. Right panels show magnifications of the framed sections shown on the left. **(B,C)** *A. fumigatus* wild type conidia were inoculated in RPMI-1640 in 96-well plates as described in the material and methods section and incubated at 37°C. After 10 h, medium was supplemented with the indicated amount of H_2_O_2_. After 2 h incubation, medium was aspirated and replaced with H_2_O_2_-free medium supplemented with resazurin. Plates were subsequently incubated at 37°C. **(B)** Photos of the depicted wells were taken after 24 h additional incubation with resazurin. **(C)** Resorufin fluorescence was documented over time with a microplate reader and plotted in the depicted graph. The error bars indicate standard deviations of three technical replicates.

Our results suggested that the fragmented mitochondrial morphology as well as disappearance of fluorescence indicates fungicidal effects of H_2_O_2_. To substantiate this hypothesis, we directly analyzed the effect of the different H_2_O_2_ concentrations on viability and survival of the mold with microscopy- and metabolism-based growth tests. As shown in Figure 3A, exposure to 1.2 and 2.4 mM H_2_O_2_ for 2 h suppressed growth of almost all *A. fumigatus* hyphae. Even after prolonged incubation for several days, clearly less than 5% of the hyphae continued to grow (not shown). However, the very few surviving hyphae rapidly overgrew the culture which significantly complicated further microscopic evaluations. Exposure of *Aspergillus* hyphae to 0.6 mM H_2_O_2_ killed approximately half of the hyphae. Due to the continuing growth of the surviving hyphae, exact numbers could not be determined. 0.3 mM H_2_O_2_ or lower did not significantly affect survival and growth. These results were in very good agreement with results obtained with a metabolism-based resazurin reduction assay (Figures 3B,C). Overall, these data demonstrate that the H_2_O_2_-induced disruption of the mitochondrial morphology correlates with hyphal death.

### Granulocytes cause non-reversible fragmentation of the mitochondrial network

**Figure 4:**
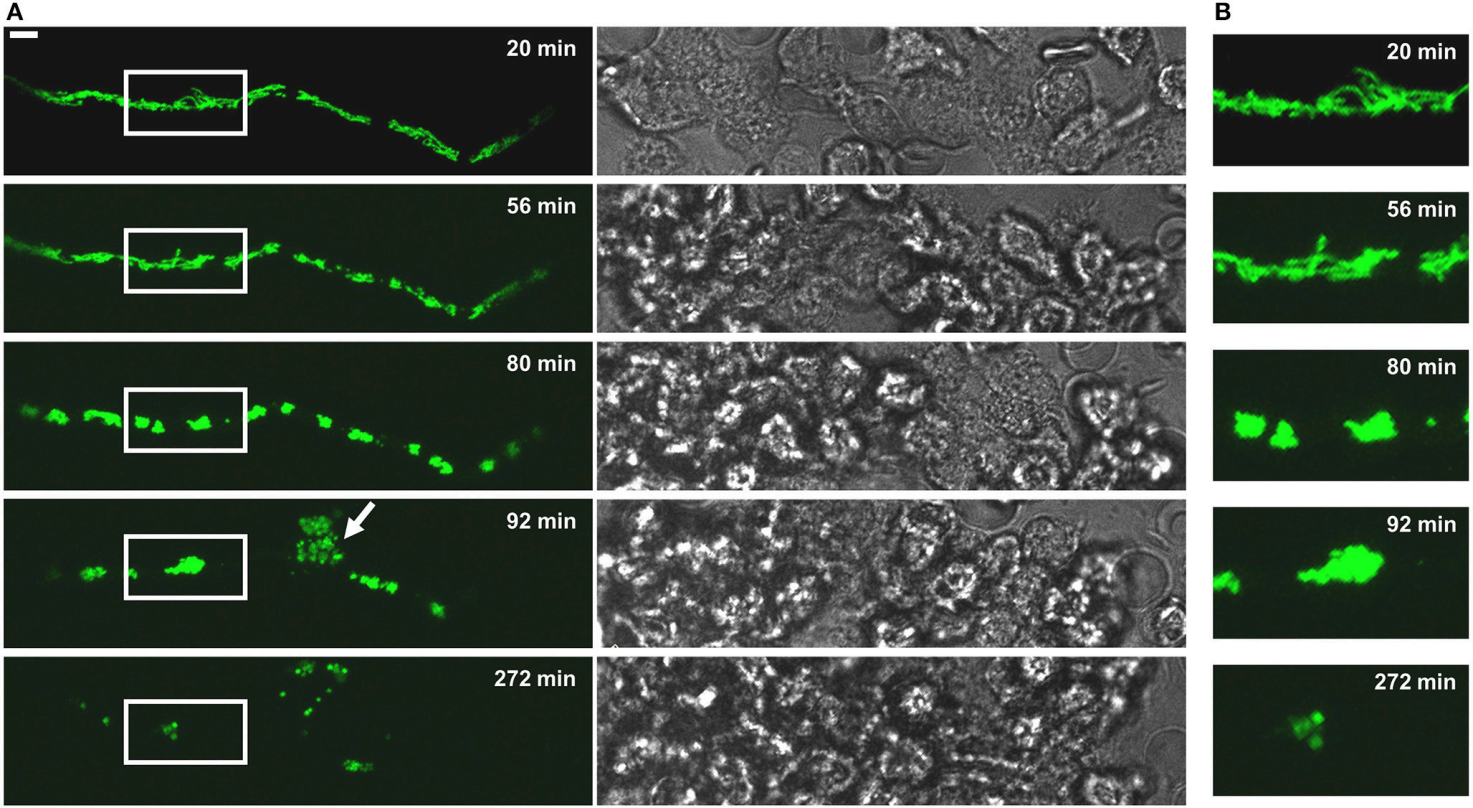
The fate of a MitoFLARE hypha after exposure to human granulocytes. *A. fumigatus* conidia expressing mitochondria-targeted GFP were inoculated in RPMI-1640 and incubated at 37°C. After 10 h incubation, granulocytes were added in medium supplemented with lipopolysaccharides (200 ng/ml final concentration). The mitochondrial morphology and fluorescence of the depicted representative hypha was subsequently documented over time with live cell microscopy. **(A)** GFP fluorescence images of optical stacks covering the entire hyphae in focus (left panels) and bright field images (right panels) were taken with a confocal laser scanning microscope at the indicated time points after addition of the granulocytes. The arrowhead indicates a cell lysis phenomenon which is occasionally seen in a minority of granulocyte-killed hyphae. The bar represents 10 μm. **(B)** Magnifications of the framed sections shown shown in **(A)**.

**Figure 5:**
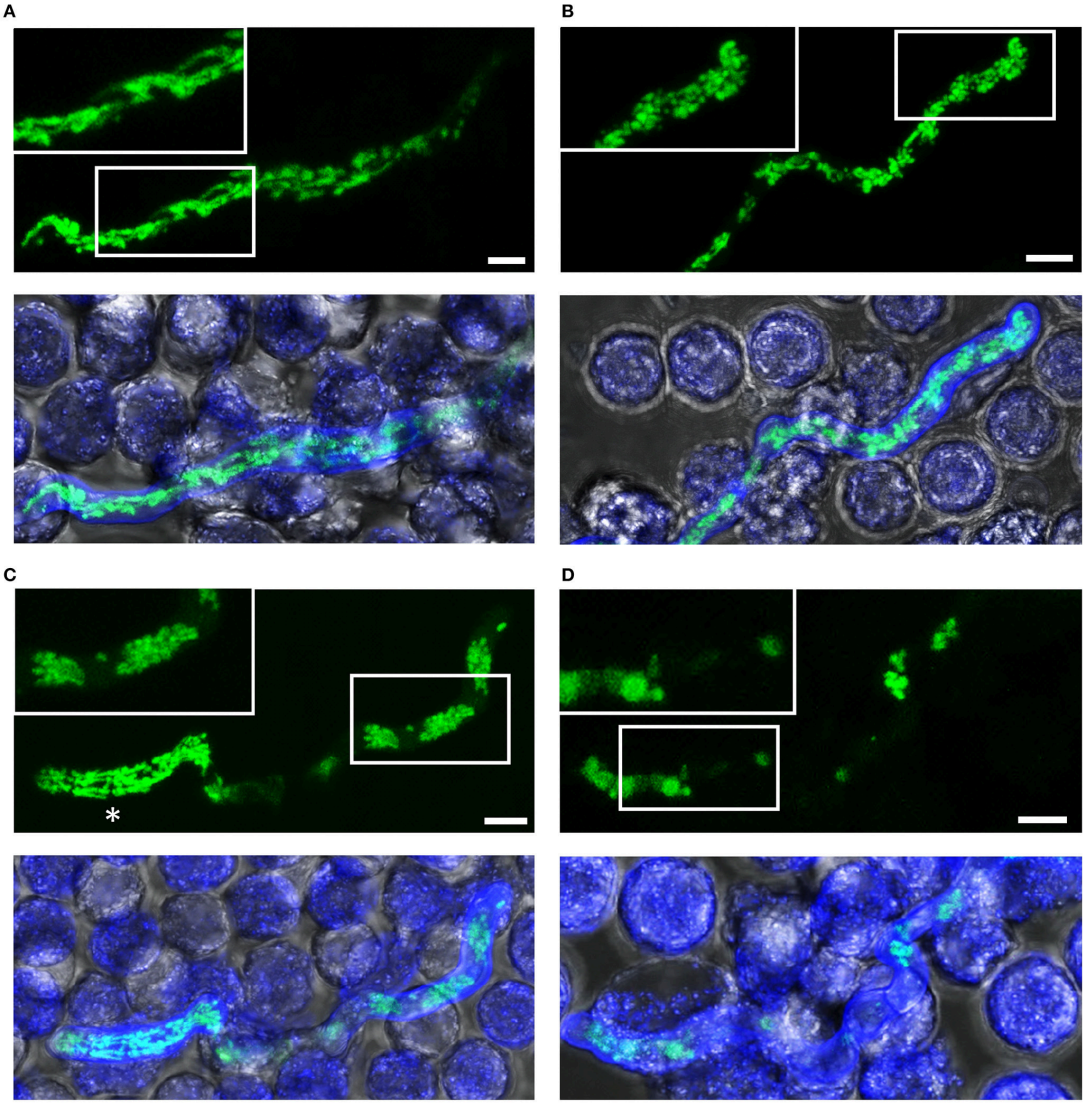
Killing of *A. fumigatus* hyphae exposed to human granulocytes for two h. *A. fumigatus* conidia expressing mitochondria-targeted GFP were inoculated in RPMI-1640 and incubated at 37°C. After 10 h, granulocytes were added. After 2 h incubation, samples were fixed and stained with calcofluor white to visualize hyphae. GFP and calcofluor white fluorescence images of optical stacks covering the entire hyphae in focus and bright field images were taken with a confocal laser scanning microscope. Upper panels show the mitochondrial morphology (green) with magnifications of the framed sections. Lower panels show overlays of bright field, GFP fluorescence and calcofluor white fluorescence. Exemplary images of hyphae or hyphal compartments with tubular **(A)**, partially tubular **(B)** or completely fragmented and clustered mitochondrial morphologies **(C)**, and of a hypha with fading GFP fluorescence **(D)**. An asterisk marks a viable compartment **(C)**, directly adjacent to a compartment with fragmented and clustered mitochondrial morphology. Bars represent 4 μm.

**Figure 6:**
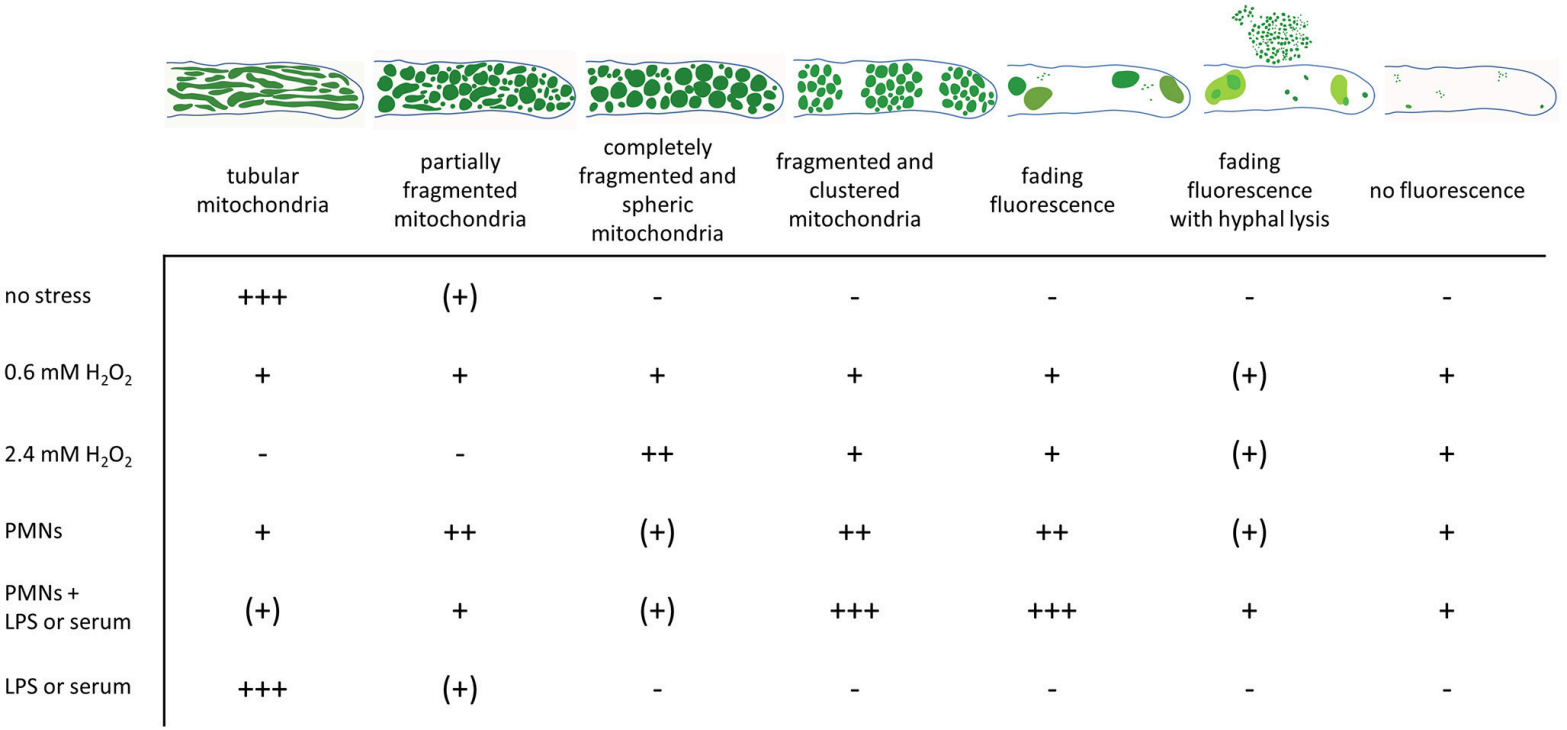
Changes in the mitochondrial morphology observed in *A. fumigatus* hyphae after exposure to hydrogen peroxide or human granulocytes. GFP-labeled mitochondria in *A. fumigatus* typically show a highly tubular and interconnected morphology. Occasionally, hyphal compartments harbor partially fragmented and partially tubular mitochondrial morphology. Exposure to fungicidal H_2_O_2_ concentrations or to granulocytes (PMNs) with antifungal activity significantly affects the mitochondrial morphology. According to our results, dying or dead hyphae expose a completely fragmented mitochondrial morphology with or without mitochondrial clustering. GFP fluorescence in such compartments tends to fade over time. Occasionally, hyphal lysis phenomenons combined with fading of the GFP fluorescence is observed. The relative frequencies of the depicted morphologies without stress or after 2 h exposure to the indicated stress is given (+++, most hyphae; ++, many hyphae; + some hyphae; (+), very few hyphae; –, no hyphae).

Neutrophil granulocytes (polymorphonuclear granulocytes, PMNs) produce reactive oxygen species (ROS), release granular content and form NETs to counteract the invasion of pathogens (Mayadas et al., 2014; Gazendam et al., 2016b,c; reviewed in Gazendam et al., 2016a). Thus, we assessed the killing efficacy of PMNs against individual *Aspergillus* hyphae. Exposure of *Aspergillus* hyphae to human granulocytes induced alterations in the mitochondrial morphology very similar to those observed in hyphae treated with fungicidal concentrations of H_2_O_2_ (Figures 4, 5 and Supplementary Videos 1–3). After 2 h exposure, many hyphae kept a tubular or partially tubular mitochondrial network (Figures 5A,B, Supplementary Video 3). Other hyphae exhibited highly fragmented and, in most cases, clustered mitochondrial morphologies (Figure 5C). In several hyphae, fluorescence was fading (<10%) or completely gone (<10%) (Figure 5D and not shown). In isolated cases, even hyphal lysis phenomenons were observed (Figure 4). An overview of the different mitochondrial morphologies that could be observed in *Aspergillus* hyphae after 2 h exposure to human granulocytes or H_2_O_2_ is shown in Figure 6. Importantly, extended video microscopy revealed that the affected hyphae failed to recover over time while those that kept a tubular or partially tubular morphology continued to grow (Supplementary Videos 4–7).

**Figure 7:**
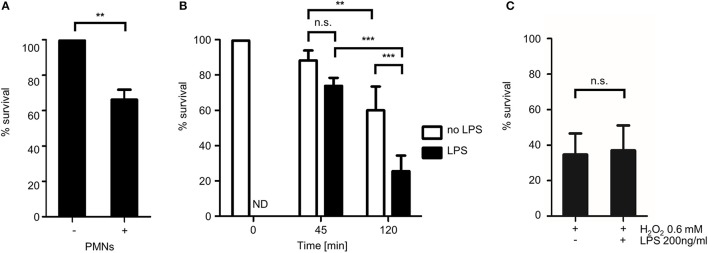
Quantitative analysis of the fungicidal activity of human granulocytes with MitoFLARE hyphae. *A. fumigatus* conidia expressing mitochondria-targeted GFP were inoculated in RPMI-1640. **(A)** When indicated, granulocytes (PMNs) were added after 10 h incubation at 37°C. **(B,C)** Granulocytes **(B)** or H_2_O_2_ (0.6 mM; **C**) were added after 10 h incubation at 37°C. When indicated, medium was additionally supplemented with lipopolysaccharides (200 ng ml^−1^ final concentration). After 2 h co-incubation **(A,C)** or the indicated time **(B)**, samples were fixed and stained with calcofluor white to visualize hyphae. The ratio of vital hyphae herein defined as hyphae with tubular or partially tubular mitochondrial morphology in more than 40% of a hyphal volume was determined as described in the material and method section. The data depicted in the graph are based on the results of one (**A**, exemplary), five **(B)** or three **(C)** independent experiments. Statistical significance (^***^p ≤ 0.001; ^**^p ≤ 0.01; n.s., not significant) was calculated with a two-tailed unpaired (assuming unequal variances) Student's *t*-test **(A,C)** or the one-way ANOVA analysis of variance with Tukey's multiple comparison post-test **(B)**. ND, not determined, the error bars indicate standard deviations.

Based on the microscopic criteria summarized in Figure 6, we quantified the impact of the granulocytes on the fungal viability. To this end, the mitochondrial morphology of in total 180 *Aspergillus* hyphae per condition after 2 h exposure to human granulocytes was evaluated directly with fluorescence microscopy. Some *A. fumigatus* hyphae may have formed septa after 10 h growth which can be sealed off, resulting in hyphae with viable and dead compartments (Figure 5C). Taking this specialty into account, we applied a similar rule as with the quantification of H_2_O_2_-induced effects and defined hyphae with tubular or partially tubular mitochondrial morphology in more than 40% of a hyphal volume as vital. On the contrary, hyphae whose mitochondrial morphology was completely fragmented or whose fluorescence was absent or fading (Figure 5D) were counted to be significantly impaired in vitality if more than 60% of the hyphal volume was affected. Based on this presumption, we found that exposure to human granulocytes caused a reduction in viability of the *Aspergillus* hyphae on an average of 30–40% after 2 h (Figure 7A).

### Lipopolysaccharides enhance granulocyte- but not hydrogen peroxide-induced mitochondrial fragmentation

It is well known that intrinsic and extrinsic mediators can modulate the antimicrobial activity of PMNs. One important extrinsic factor that activates neutrophil granulocytes are lipopolysaccharides (LPS) of gram negative bacteria. To test whether LPS can boost the antifungal activity of human granulocytes against *Aspergillus* hyphae, we exposed *Aspergillus* hyphae to human granulocytes that were or were not additionally stimulated with ultra-pure LPS from *Escherichia coli* K12. As shown in Figure 7B, LPS significantly increased the impact of the human granulocytes on the mitochondrial morphology. The spectrum of morphological alterations that could be observed in affected hyphae exposed to LPS-stimulated granulocytes was identical compared to the one observed in hyphae exposed to non-LPS-stimulated granulocytes. Importantly, LPS alone did not affect the mitochondrial morphology of *Aspergillus* hyphae nor did it increase the antifungal activity of H_2_O_2_ (data not shown and Figure 7C).

### Serum enhances the killing efficacy of granulocytes against *Aspergillus* hyphae

**Figure 8:**
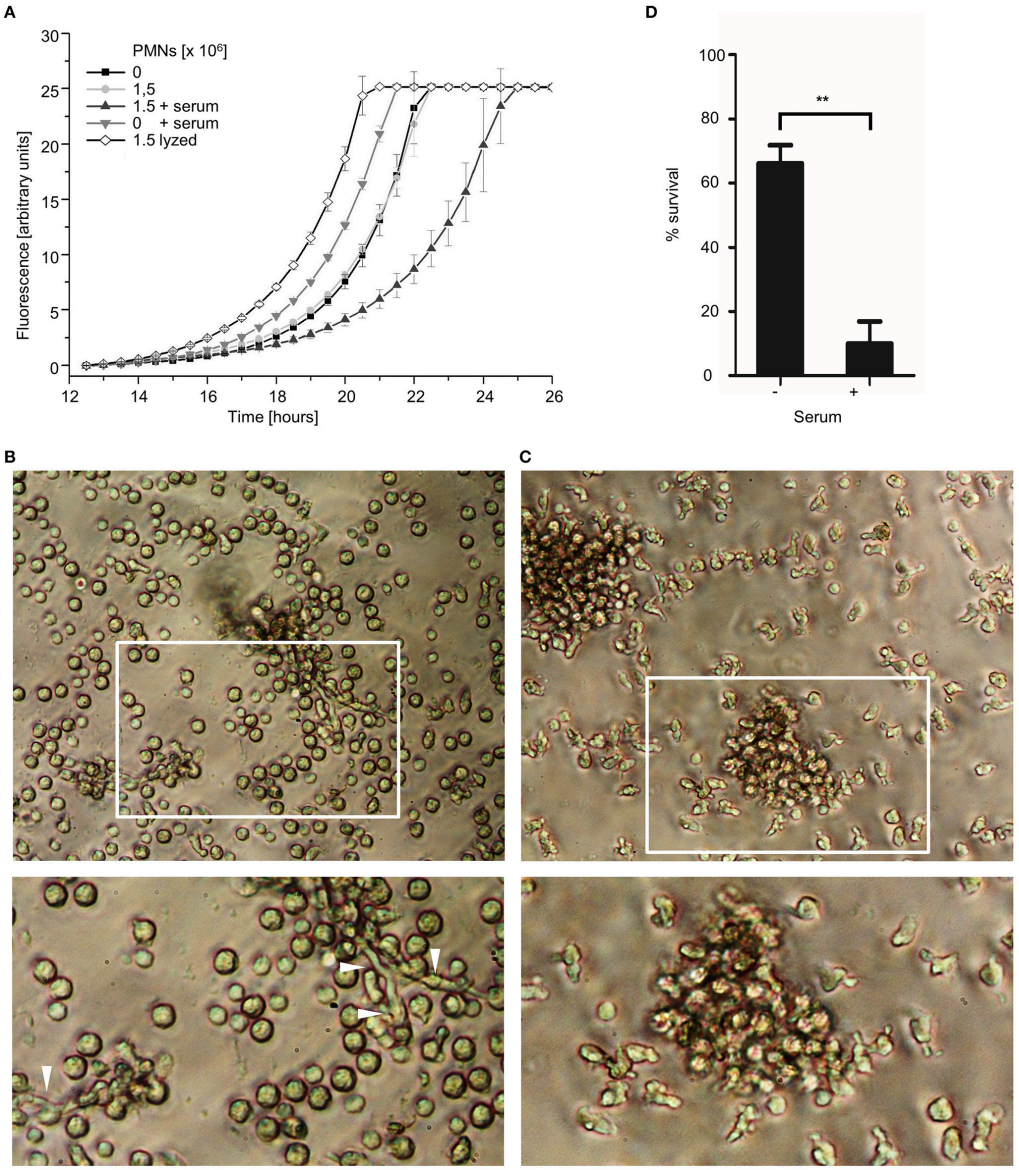
Serum enhances the antifungal killing activity of human granulocytes. **(A)** *A. fumigatus* wild type conidia **(A–C)** or *A. fumigatus* conidia expressing mitochondria-targeted GFP **(D)** were inoculated in RPMI-1640 and incubated at 37°C. After 10 h, human granulocytes were added. When indicated 10% (v/v) human serum or an equal volume of medium was added 30 min prior to addition of granulocytes. **(A)** After 2 h incubation, granulocytes (PMNs) were lyzed with ice-cold ddH_2_O for 30 min followed by the addition of *Aspergillus* minimal medium supplemented with resazurin. Controls were treated likewise but in case of the lyzed-granulocytes control (lyzed) with ice-cold ddH_2_O supplemented with lyzed granulocytes. Resorufin fluorescence was documented at 37°C over time with a microplate reader and plotted in the depicted graph. The error bars indicate standard deviations. **(B,C)** Exemplary bright field images of granulocytes and hyphae (arrowheads) after 2 h co-incubation with **(C)** or without **(B)** serum. Magnifications of the framed sections are shown below. **(D)** After 2 h co-incubation, samples were fixed and stained with calcofluor white to visualize hyphae. The ratio of vital hyphae herein defined as hyphae with tubular or partially tubular mitochondrial morphology in more than 40% of a hyphal volume was determined as described in the material and method section. The data depicted in the graph are based on the results of three independent experiments. Statistical significance (^**^p ≤ 0.01) was calculated with a two-tailed unpaired (assuming unequal variances) Student's *t*-test. The error bars indicate standard deviations.

It was recently reported that serum enhances the antifungal activity of granulocytes against *Aspergillus* hyphae (Gazendam et al., 2016c). In this study, a metabolism-based colorimetric MTT (3-(4,5-dimethylthiazol-2-yl)-2,5-diphenyltetrazolium bromide) reduction assay was used to measure the antifungal activity of the granulocytes. In good agreement with these results, we observed a similar serum-dependent decrease in growth of *Aspergillus* hyphae after exposure to human granulocytes by using a metabolism-based resazurin reduction assay (Figure 8A). Interestingly, serum appeared to not only increase the visual adherence of granulocytes to *Aspergillus* hyphae as reported by Gazendam et al. (2016c), but additionally induced a general change of the immune cells' shape which was independent of direct contact to hyphal structures (Figures 8B,C). To clarify whether serum improves the fungicidal activity of the immune cells, we evaluated the mitochondrial morphology of *Aspergillus* hyphae 2 h after exposure to human granulocytes in the presence or absence of human serum. As shown in Figure 8D, serum drastically increased the killing efficacy of the granulocytes. While granulocytes killed approximately 30% of the hyphae without serum, approximately 90% were killed with serum (Figure 8D). Again, serum alone did not affect the mitochondrial morphology of *Aspergillus* hyphae (Supplementary Figure 3).

## Discussion

Immune cells exert different antimicrobial activities to control and fight off opportunistic infections. In case of neutrophil granulocytes these activities include the generation of ROS, the release of intracellular granules with myeloperoxidase, proteases and ion-sequestering proteins such as calprotectin, lactoferrin and the formation of NETs (Clark et al., 2016; Gazendam et al., 2016a). Importantly, the individual contributions of these different antimicrobial activities to growth inhibition and inactivation of fungal pathogens greatly differ and additionally depend on the fungal species and morphotype. For example, azurophilic granules were recently shown do exert a fungicidal activity against *A. fumigatus* hyphae but not against *A. fumigatus* conidia (Gazendam et al., 2016c). NETs, however, have the potential to contribute to killing of *A. fumigatus* hyphae at later time-points (Bruns et al., 2010), but the major effect against hyphae appears to be of fungistatic nature (Bruns et al., 2010; McCormick et al., 2010; Gazendam et al., 2016c). The newly introduced mitochondrial morphology and fluorescence-based killing assay described herein allows for the evaluation of antifungal activity on a per-cell basis and specifically aims on early detection and quantification of fungicidal effects. Because of this, it greatly complements existing methodological approaches that quantify the growth rate of hyphae after exposure to immune cells (e.g., as determined with colorimetric tetrazolium reduction assays) to discriminate fungicidal from fungistatic effects.

We demonstrated that the morphological alteration of the mitochondria caused by H_2_O_2_ or human granulocytes is an early sign of fungicidal cell damage. As expected, addition of LPS significantly enhanced the effect of granulocytes but not of H_2_O_2_ on the mitochondrial morphology and fluorescence of hyphae. Similar, by utilizing the MitoFLARE hyphae, we showed that serum-opsonized hyphae are more efficiently killed by human granulocytes compared to non-opsonized hyphae. These results illustrate that MitoFLARE hyphae are suitable to detect discrete differences in the antifungal activity of immune cells. Importantly, the affected hyphal compartments did not reconstitute tubular mitochondrial morphology nor did they continue to grow after complete fragmentation (Supplementary Videos 4–7). Further, video microscopy of these hyphae revealed that the GFP fluorescence fades over time, a sign that was previously applied to quantify killing of *Aspergillus* conidia (Jhingran et al., 2012, 2016; Espinosa et al., 2014; Heung et al., 2015; Brunel et al., 2017).

Our approach has significant advantages compared to available assays that are solely based on fading of cytosolic fluorophores (Jhingran et al., 2012, 2016; Espinosa et al., 2014; Heung et al., 2015; Brunel et al., 2017). Depending on the fungicidal activity, the half-life of a fluorophore can significantly vary (Heung et al., 2015). For example, while severe cell lysis phenomenons as caused by echinocandin antifungals will result in extinction of cytosolic fluorescence within minutes (Dichtl et al., 2015), other fungicidal activities such as induction of apoptosis or exposure to ROS may not immediately affect the fluorophores (Shlezinger et al., 2017 and this study). This will be critical if the growth rate of the pathogen is significantly faster than the fading of the fluorescence or fungicidal activities with different manifestations compete with each other. It was reported that the fluorescence of DsRed-expressing *Escherichia coli* digested in *Dictyostelium discoideum* phagolysosomes fades with a half-life of 45 min (Maselli et al., 2002). However, *A. fumigatus* hyphae extend with a hyphal tip velocity of 6–8 μm per minute accompanied by regular hyphal branching in mammalian cell culture media (Ellett et al., 2017). This will presumably hamper any quantitative analysis of killing because the remaining surviving hyphae, in marked contrast to resting conidia, will rapidly overgrow. The MitoFLARE hyphae showed significant fragmentation of the mitochondrial morphology in dying hyphae within less than 2 h after addition of the granulocytes. Significant fading of the GFP fluorescence was observed only in a small minority of the hyphae at this time point. This indicates that MitoFLARE hyphae will report killing faster than FLARE hyphae.

In summary, we technically extended the concept of FLARE that correlates death of fungi and fading fluorescent proteins with an additional and independent readout that is affected mitochondrial morphology. We demonstrated that MitoFLARE can be utilized to quantify the killing efficacy of granulocytes against *A. fumigatus* hyphae. In addition, we demonstrated that MitoFLARE reports death of hyphae in a timely manner, making it an excellent tool for video microscopy of host pathogen interactions *in vitro*. Applications *in vivo* are also conceivable but, so far, have not been tested. We propose that MitoFLARE will be a helpful tool to evaluate and characterize fungicidal effects against *Aspergillus* hyphae in future studies.

## Author contributions

JW conceived the study; DR, VB, and JW designed the experiments; DR and VB performed the experiments; DR, VB, and JW analyzed experimental data, wrote the manuscript and prepared the figures.

### Conflict of interest statement

The authors declare that the research was conducted in the absence of any commercial or financial relationships that could be construed as a potential conflict of interest.

## Supplementary material

Supplementary Figure 1 Hydrogen peroxide susceptibility and hydrogen peroxide-induced mitochondrial fragmentation does not depend on non-homologous end joining in *A. fumigatus*. **(A,B)** 2 × 10^5^ conidia of the *A. fumigatus* wild type strain D141 or of its non-homologous end joining-deficient derivative AfS35 were spread on an AMM agar plates in triplicates. 50 μl 300 mM (left) or 100 mM (right) H_2_O_2_ were filled in punch holes and the agar plates were incubated at 37°C. **(A)** Exemplary images were taken after 42 h. **(B)** The diameters of the 300 mM inhibition zones were measured after 42 h incubation and plotted in the bar graph. Statistical significance (n.s., not significant) was calculated with a two-tailed unpaired (assuming unequal variances) Student's *t*-test. The error bars indicate standard deviations. **(C)** Conidia of the D141 strain expressing mitochondria-targeted GFP were inoculated in AMM. After 10 h incubation at 37°C, medium was supplemented with 3 mM H_2_O_2_. The mitochondrial morphology of the depicted hyphae was documented over time with confocal laser scanning microscopy. An exemplary bright field image (left) and time-lapse GFP fluorescence images of optical stacks covering the entire hyphae in focus after 0, 30 and 50 min exposure (green; middle and right) are depicted. Bars represents 5 μm.

Supplementary Figure 2 Persisting cytosolic GFP fluorescence after exposure to fungicidal hydrogen peroxide concentration. **(A,B)** *A. fumigatus* conidia expressing mitochondria-targeted GFP **(A)** or cytosolic GFP **(B)** were inoculated in RPMI-1640 and incubated at 37°C for 10 h. When indicated, medium was supplemented with 2.4 mM H_2_O_2_. After 2 h additional incubation, samples were fixed and analyzed with fluorescence microscopy. Exemplary bright field (lower panels) and GFP fluorescence (upper panels) images of optical stacks covering the entire hyphae in focus were taken with a confocal laser scanning microscope. Bars represent 5 μm.

Supplementary Figure 3 Human serum does not affect the mitochondrial morphology of *A. fumigatus* hyphae. *A. fumigatus* conidia expressing mitochondria-targeted GFP were inoculated in RPMI-1640 and incubated at 37°C. After 9.5 h incubation, medium was supplemented with 10% (v/v) human serum or an equal volume of medium. After 2.5 h co-incubation, samples were fixed and stained with calcofluor white to visualize hyphae. The ratio of vital hyphae herein defined as hyphae with tubular or partially tubular mitochondrial morphology in more than 40% of a hyphal volume was determined as described in the material and method section. The data depicted in the graph are based on the results of three independent experiments. Statistical significance (^**^*p* ≤ 0.01) was calculated with a two-tailed unpaired (assuming unequal variances) Student's *t*-test. The error bars indicate standard deviations.

Supplementary Videos 1-3 Exemplary videos of *A. fumigatus* hyphae that were killed (**Videos 1, 2**) or not killed (**Video 3**) by human granulocytes stimulated with LPS. *A. fumigatus* conidia expressing mitochondria-targeted GFP were inoculated in RPMI-1640 and incubated at 37°C. After 10 h incubation, granulocytes were added in medium supplemented with lipopolysaccharides (200 ng/ml final concentration). GFP fluorescence of optical stacks covering the entire hyphae in focus and bright field were then documented over time with live cell microscopy. GFP fluorescence (GFP) and overlay of bright field and GFP fluorescence (overlay) are available as separate videos. Time lapse images from **Video 2** are also depicted in Figure 4.

Supplementary Videos 4-7 Exemplary videos of serum-opsonized *A. fumigatus* hyphae that were partially or completely killed by human granulocytes. *A. fumigatus* conidia expressing mitochondria-targeted GFP were inoculated in RPMI-1640 and incubated at 37°C. After 9.5 h, 10% (v/v) human serum was added followed by 30 min incubation at 37°C. Granulocytes were added and co-incubated for 2 h. Granulocytes were subsequently lyzed with ice-cold ddH_2_O for 30 min followed by the addition of *Aspergillus* minimal medium. GFP fluorescence of optical stacks covering the entire hyphae in focus and bright field were then documented over time with live cell microscopy. GFP fluorescence (GFP) and overlay of bright field and GFP fluorescence (overlay) are available as separate videos.
