# Supplementary figures and images for: Mitochondrial Fragmentation in Aspergillus fumigatus as Early Marker of Granulocyte Killing Activity
Source: Front Cell Infect Microbiol. 2018 May 14;8:128. doi: 10.3389/fcimb.2018.00128 (PMC5960683; doi:10.3389/fcimb.2018.00128)

# Supplementary Figure 1

**A**

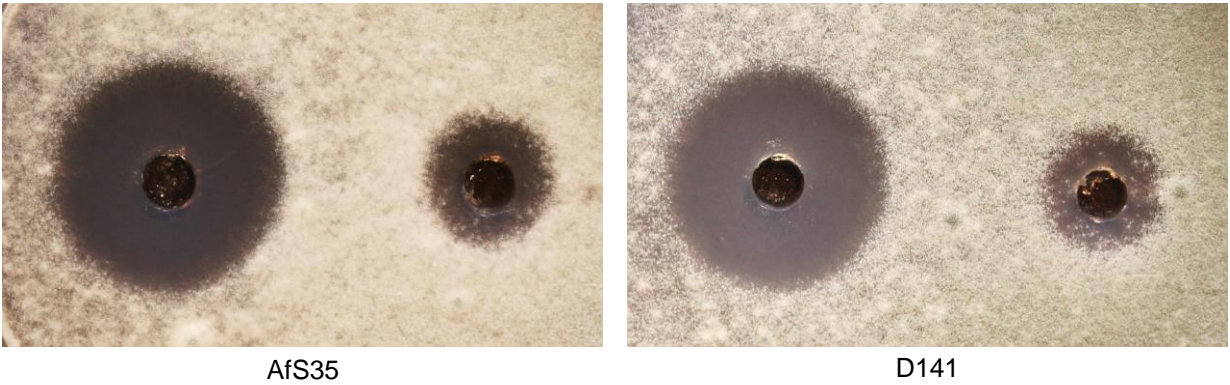

**B**

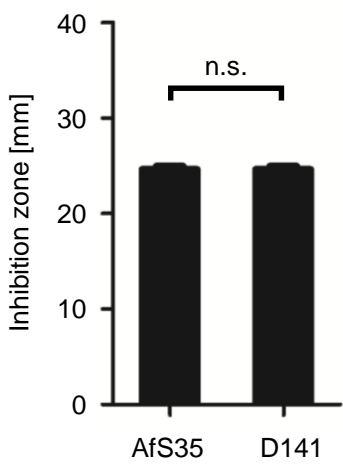

**C**

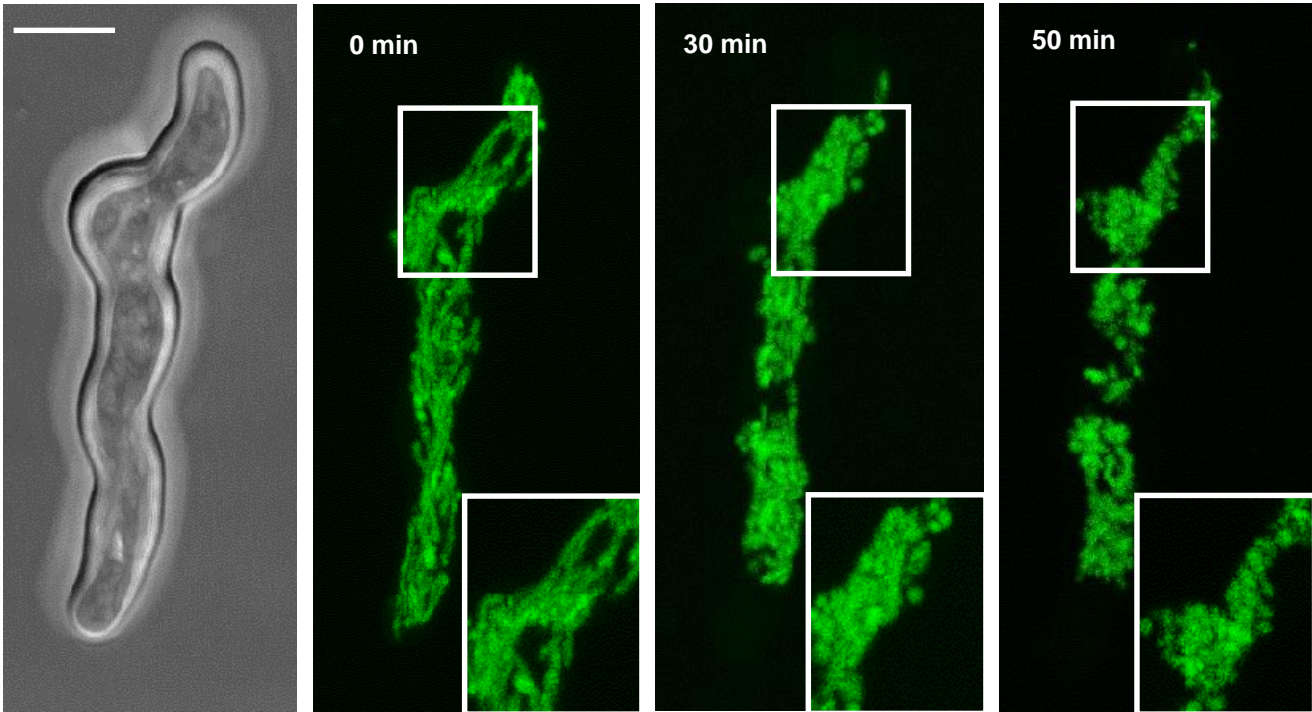

Supplementary Figure 2

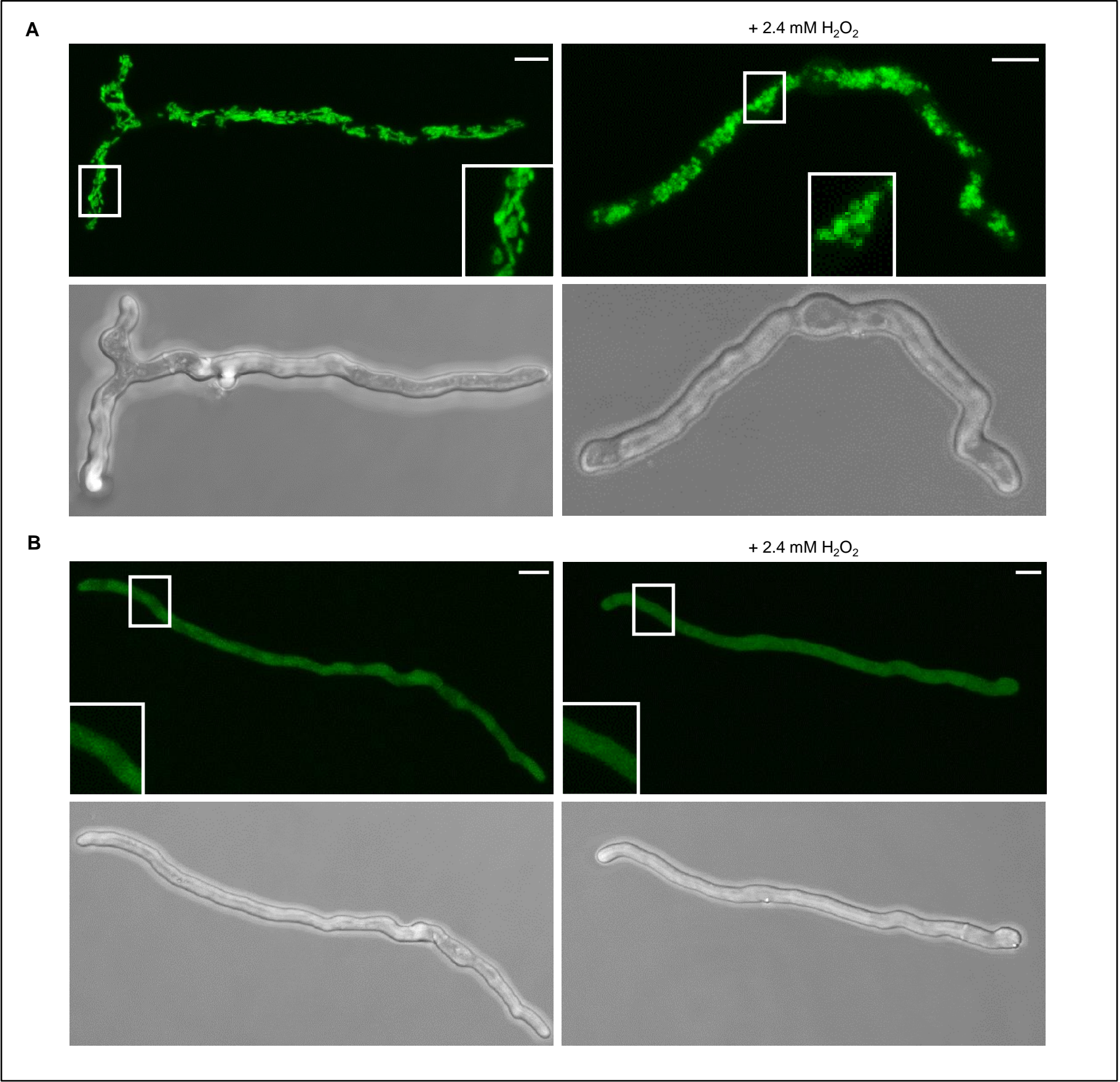

# Supplementary Figure 3

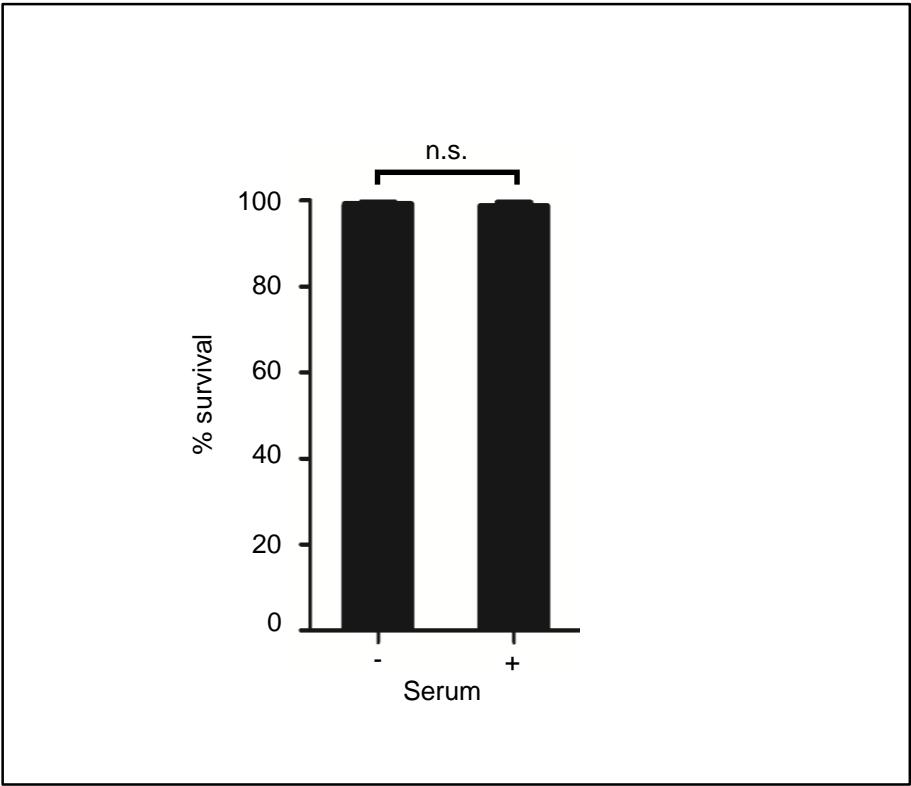

Supplement: Supplementary Figure 1 — Hydrogen peroxide susceptibility and hydrogen peroxide-induced mitochondrial fragmentation does not depend on non-homologous end joining in A. fumigatus. (A,B) 2 × 10^5^ conidia of the A. fumigatus wild type strain D141 or of its non-homologous end joining-deficient derivative AfS35 were spread on an AMM agar plates in triplicates. 50 μl 300 mM (left) or 100 mM (right) H2O2 were filled in punch holes and the agar plates were incubated at 37°C. (A) Exemplary images were taken after 42 h. (B) The diameters of the 300 mM inhibition zones were measured after 42 h incubation and plotted in the bar graph. Statistical significance (n.s., not significant) was calculated with a two-tailed unpaired (assuming unequal variances) Student's t-test. The error bars indicate standard deviations. (C) Conidia of the D141 strain expressing mitochondria-targeted GFP were inoculated in AMM. After 10 h incubation at 37°C, medium was supplemented with 3 mM H2O2. The mitochondrial morphology of the depicted hyphae was documented over time with confocal laser scanning microscopy. An exemplary bright field image (left) and time-lapse GFP fluorescence images of optical stacks covering the entire hyphae in focus after 0, 30 and 50 min exposure (green; middle and right) are depicted. Bars represents 5 μm. [file Presentation_1.PDF]
